# Patients’ views on usefulness and effects of a risk communication tool for cardiovascular disease: a qualitative analysis

**DOI:** 10.1186/s12875-024-02279-7

**Published:** 2024-02-03

**Authors:** Anders Elkær Jensen, Jens Søndergaard, Niels Kristian Kjær, Jesper Bo Nielsen

**Affiliations:** Research Unit of General Practice, Department of Public Health, University of Southern Denmark, Campusvej 55, Odense, 5230 Denmark

**Keywords:** Qualitative, Patient interview, Risk communication, Visual communication, Cardiovascular disease, General practice, Primary sector, Family medicine

## Abstract

**Background:**

Failing to comprehend risk communication might contribute to poor treatment adherence. Using hypertension as a case, we investigated how a risk communication tool for patients with an elevated risk of cardiovascular disease was perceived.

**Methods:**

As part of a large project featuring a randomised controlled trial in a general practice setting in the Region of Southern Denmark, we conducted a semi-structured individual interview study. The study included patients with hypertension who had used an intervention comprising a visual and dynamic cardiovascular risk communication tool, along with receiving recurring emails providing advice on a healthy lifestyle. The analyses were based on Malterud’s Systematic Text Condensation.

**Results:**

This article focuses solely on the results of the interview study, which comprised a total of 9 conducted and analysed interviews. The IT setup had a major impact on adherence to the intervention. A positive impact was found when the IT setup was perceived as easy to use and accessible, while a negative impact was noted when it malfunctioned. The intervention increased patients’ self-reported insight into risk of cardiovascular disease. Patients reported the intervention and their risk of cardiovascular disease to become less important to them when they had more severe comorbidities. The involved health professional was very important for treatment adherence when communicating risk visually. Patients expressed trust in their general practitioners, and the general practitioners’ attitudes toward the intervention affected patients’ perceptions of its usefulness. While the informants reported an increased awareness of their risk of cardiovascular disease, none of them felt more concerned.

**Conclusions:**

Patients reported an increase in their perceived insight into the risk of cardiovascular disease but not an increased concern. Our findings align with previous studies emphasizing the importance of patients’ motivation as well as risk perception for adherence. General practitioners have an important role when implementing new tools for patients.

## Introduction

Understanding patients’ perceptions of the communication tools is crucial for enhancing perceived usefulness when implementing them.

Currently, Danish General Practitioners (GPs) predominantly use SCORE chart to communicate cardiovascular disease (CVD) risk, relying more on numerical values than visual aspects [1]. Many patients, however, struggle to connect SCORE chart information to their individual health and lifestyle [2].

Across all countries, a portion of the population exhibits low health literacy, defined as:

“…people’s knowledge, motivation and competences to access, understand, appraise, and apply health information in order to make judgements and take decisions in everyday life concerning healthcare, disease prevention and health promotion to maintain or improve quality of life during the life course [3]”.

In Denmark, this fraction is approximately 20%, while other European countries report up to 40% with low health literacy [4–6].

Low health literacy may contribute to the fact that only one-third of Danish patients diagnosed with hypertension successfully achieve treatment goals outlined in national guidelines [1, 7, 8]. Health literacy plays a crucial role in behavioural change and adherence to such changes, as supported by various behavioural change theories [9, 10].

The Self-Determination Theory (SDT) emphasizes autonomy, relatedness and competence as key themes for sustained intrinsic motivation in behavioural change [11]. Autonomy involves the choice to change lifestyle, competence relates to the belief in one’s abilities and relatedness addresses the sense of community in the pursuit og lifestyle change [11].

The Theory of Planned Behaviour (TPB) highlights the significance of the initial presentation of an intervention. Attitudes toward changing health behaviour correlate with perceived control and subjective norms [12]. To alter patients’ subjective perception of the normal, introducing visual communication and instilling belief in their ability to succeed in behavioural change are crucial.

Contrary to common belief, evidence suggests that GP’s attitudes toward nonadherence have minimal influence on patients’ adherence [13]. The Transtheoretical Model of Health Behaviour Change (TTM) provides a foundation for strengthening patients’ adherence to treatment, particularly in the maintenance stage, preventing relapse into previous lifestyles [14].

For interventions to be effective in supporting patients in behavioural change, they must align with SDT, TPB, and TTM. Increasing patients’ health literacy is essential for improving competences and altering perceptions of normalcy and potential behavioural change effects. While the decision to change behaviour rests with the patient, support is necessary both in making the decision and adhering to the change. The theories SDT, TPB, and TTM underpin the believe that the chosen intervention can support patients in behavioural change and enhance treatment adherence.

We investigated how a dual intervention consisting of a visual risk communication tool for CVD and recurrent emails was perceived by patients and it’s potential to improve adherence to planned treatment in primary care. The visual communication tool was intended to enhance patients’ competences by improving their understanding of CVD risk. It is essential for a wider use of the intervention to learn about it from the patients’ perspective [8]. Our inquiry focuses on whether patients perceive the intervention as relevant and useful in managing their risk of CVD.

## Aim

The study aims to shed light on the participants’ experience with the visual risk communication tool through four research questions:

- How does the intervention affect the patients’ perceived insight into their own CVD risk?
- Do the patients perceive the intervention as supportive of adhering to a healthy lifestyle?
- Does the intervention affect patients’ perceived health?
- Does the intervention appear useful in practice and how does it affect the patients’ motivation?

## Methods

This paper follows the COREQ 32-item checklist to assure that agreed standards for reporting qualitative research is maintained [15].

### Research team

All interviews were carried out by PhD-student AEJ, who is a male medical doctor with 9 years of clinical experience from hospitals and general practice. Researchers JBN, JS and NKK are all senior researchers and have extensive research experience from both quantitative and qualitative research within general practice. Furthermore, JS and NKK have decades of experience as General Practitioners.

There were no formal and/or planned conversations between AEJ and the informants prior to the interviews. The informants were explained that AEJ was the project leader and a medical doctor but was otherwise not informed of any personal goals or reasons of the interviewer.

### Study design

This study was a qualitative study consisting of semi-structured interviews. The study was part of a larger project with a randomised controlled trial, where a dual intervention of an online risk communication tool and biweekly recurrent emails was tested on patients with the diagnosis of hypertension. An online questionnaire was sent to the participants by email through REDCap, at the beginning of the RCT, after 6 months and after 12 months. The project was set in general practice in the Region of Southern Denmark and the purpose was to see if the intervention could lower patients’ blood pressure through increased understanding of their own CVD risk. This article only reports on results from the qualitative interviews.

The data analysis was based on systematic text condensation according to Malterud, a method repeatedly used in qualitative studies within general practice [16, 17].

**Fig. 1:**
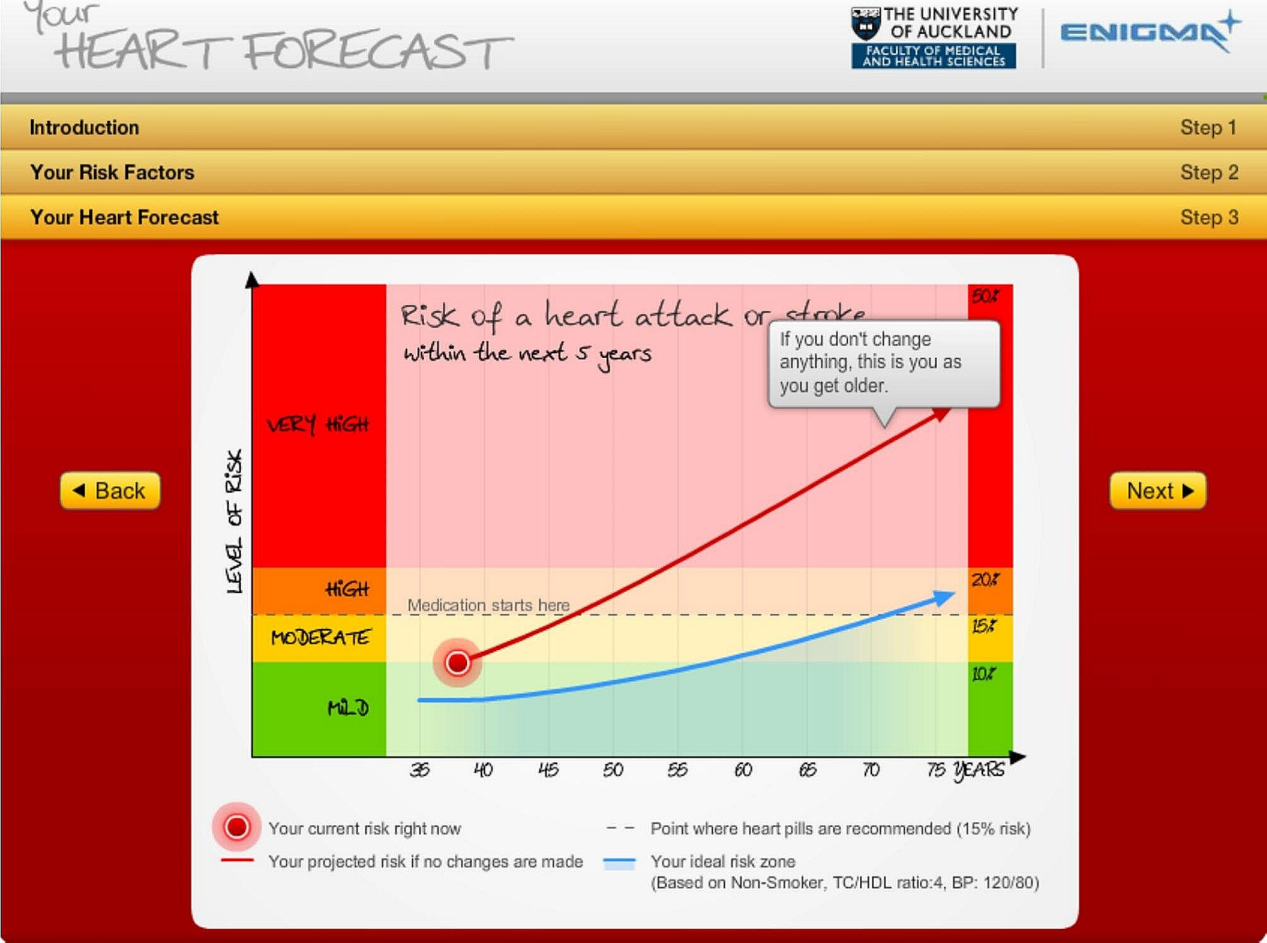
The visual risk communication tool: “Your Heart Forecast”. All text was translated to create the Danish version of the tool used in the randomised controlled trial [20]

Informants were chosen from the intervention group of the randomised controlled trial where the communication tool was used [18]. The communication tool used was “Your Heart Forecast” and a screen print of it can be seen in Fig. 1. Your Heart Forecast is an online, interactive and dynamic software visualising and predicting a patient’s present absolute risk of CVD, the predicted risk until age 75, as well as the influence of changing several lifestyle-related risk factors [19].

The tool is intended to be introduced to patients by their GP and with subsequent online and independent patient access to the program. In addition to the visual communication tool, the intervention consisted of recurring emails with advice on how to live a healthy lifestyle. A protocol article has been published separately [18].

Nine informants were purposively chosen to cover different traits (geographical habitation, sex, age, educational level, and occupational status) in the background population. The informants had participated in the study between 6 and 12 months at the time of their interview. No registration was done of how frequently participants had used the visual software.

All informants were contacted by phone and all interviews were carried out as phone interviews. Two of the contacted trial participants did not want to participate in the interview study and were immediately replaced by two other informants, maintaining the original purposive diversity. After 7 interviews, no additional information seemed to appear. The interviews then circled the same topics without new ones being addressed. It was subsequently assumed that further interviews would not give any additional information and therefore information saturation was assumed after a total of 9 interviews.

All interviews were done while the interviewer was alone in the workplace office. All informants but two were reached at home.

All informants were at the initial contact offered to do the interview right away or reschedule to a more convenient time.

The same interview guide was used for all interviews to make sure that the predefined topics were covered (Table 1), without excluding the possibility of exploring themes brought up by the informants. Predefined topics were addressed when they occurred naturally in the conversation during the interview and were not picked in any specific sequence.

All interviews were audio-recorded in one take and no repeated interviews were made. Interviews took between 12 and 20 min. The first author carried out all interviews and made all transcriptions. The informants were not given the opportunity to comment on the transcriptions. Interviews were done from September 2020 until August 2021. The interviews were done in Danish. The results were translated into English after the analysis was done. All translation were done by the first author and the other authors, independently agreed on translation.

### Analysis and findings

**Fig. 2:**
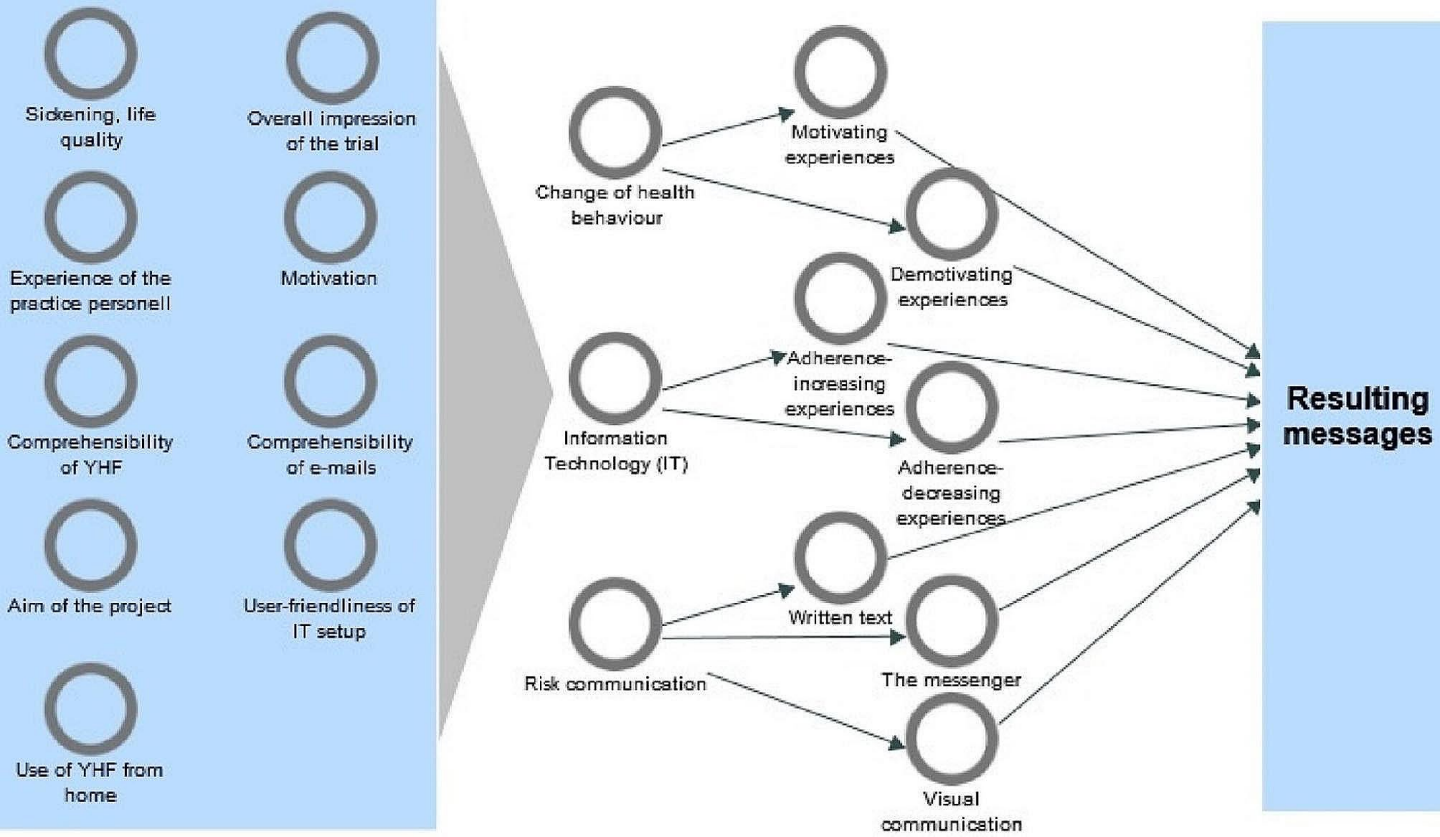
Code tree of the four steps of analysis

Data were analysed using NVivo12. Two authors (AEJ and JBN) independently read all transcripts and subsequently agreed on the relevant focus areas to be used. The 4 steps of analysis and corresponding focus areas/codes can be seen in Fig. 2. In step 1 of Systematic Text Condensation [16], the topics of the interview guide were identified but were also supplemented with new recurring topics from the interviews. In step 2 the conversational topics were translated into 3 fitting focus areas which made the basis for the condensation in step 3, where meaningful sub-areas were identified. Eventually, in step 4 we synthesised resulting messages from the data of our study.

Informants were not shown the final findings before publishing.

**Table 1:** Core topics in the interview guide

Topics	Example of introduction
Overall impression of the trial	Would you try to describe your experience of participating in the trial?
User-friendliness of the IT setup	How has it been to use the questionnaires and the biweekly emails?
Thoughts of Your Heart Forecast	What do you think of the information you got when using the program Your Heart Forecast?
Thoughts of biweekly emails	What do you think of the information you got through the biweekly emails?
How patients experienced the staff	How did you experience the doctors and nurses who participated in the study, using the program Your Heart Forecast and its figures?
Use of Your Heart Forecast from home and discussion with relatives	How did you experience being able to access your risk profile from home and together with your relatives?
Concerns	How did it affect you, that we created an increased attention/awareness of your risk of illness?

## Results

### Informant characteristics

Informants were chosen to represent different ages relevant for CVD, educational levels, and geographic regions. For details on informant characteristics, see Table 2.

**Table 2:** Informant characteristics

Informant number	Age	Sex	Education	Job-status	Part of region*
1	75	F	Elementary school	Retired	4
2	39	M	Short higher education (1–4 years)	Absent (illness)	1
3	71	M	Short higher education (1–4 years)	Working	2
4	73	F	Short higher education (1–4 years)	Retired	4
5	70	M	Elementary school	Retired	3
6	74	M	Short higher education (1–4 years)	Retired	3
7	48	F	Elementary school	Working	2
8	52	F	Vocational education	Working	2
9	54	F	Long higher education (5 + years)	Working	1

*The region of Southern Denmark is divided in to four parts regarding healthcare. The four parts each have one hospital with emergency functions and other minor hospitals/clinics with elective functions. Two of the four parts are more rural than the other two. Names of the parts of the region have been removed to anonymise the data

### Analysis outcome

After conducting 7 interviews no new topics emerged. After a total of 9 interviews, the authors concluded that additional interviews would not lead to additional information and assumed that information saturation was reached. From the analysis of the 9 transcripts, we identified 3 focus areas: Change in health behaviour, Information Technology (IT) and Communication. For all three focus areas, we identified sub-areas before synthesising the resulting messages. Table 3 summarises our results. To illustrate the resulting messages, citations from the interviews have been put in the text in italics.

#### Change in health behaviour

The continuously reminders had inconsistent effects on the perceived motivation. Some of the informants reported the reminders as motivating because they offered good advice or assured the informants that what they did already, was correct. Other informants did not see any gain from the reminders because they thought to know the contents already. Continuously reminders were in no case perceived as a negative influence.

Informant number 8:

“So, you just get that reminder that you do not have to just fall down on the couch and rest, uh. So, the questions have been nice and easy, and precisely a reminder that, yes, it could well be that you just have to remember a little more vegetables and go for a walk, and just all those things, so you just get reminded once in a while.”

Two other motivating factors were the feeling of an increase in insight into their CVD risk and a personal health gain. Informants who felt an increase in insight into CVD risk were eager to use their new knowledge and those who either saw an option to gain or felt an ongoing health improvement were motivated to proceed or persevere.

Informant number 4:

So … I probably became a little more aware that family disease patterns also is a factor. Now I lost my dad pretty early from stroke so … it was kind of like well yeah, so it’s something in the family, so I should probably take it seriously then.

One informant felt highly motivated by having the autonomy to use the intervention when it fitted the packed schedule and one informant reported motivation from feeling closer to the onset of illness, thus making it more relatable.

Informant number 8:

The fact that you have just been able to do it, whenever you think, now you had just time for it and you sat down on the couch in the evening and then it was easy to just take them on the phone, so you did not have to go in and start a PC up and uh, so that way… I did it all on the phone, pretty much, I only think I’ve been on my PC once or twice.

A demotivating obstacle to adhere to the intervention was competing illnesses. Patients reported that focus on the trial was diminished when another competing illness occurred. The high blood pressure and their risk of CVD would become less important because a more imminent threat to their life quality emerged.

Other demotivating factors were non-functioning IT solutions and non-committed health care personnel, but these are further elaborated below under 2. IT and 3. Communication

Informant number 7:

I simply have no surplus to it because I have fibromyalgia and we struggle enough to keep me up there, and my work and stuff like that.

#### IT

The IT solutions discussed in the interviews were the Your Heart Forecast tool, the bi-weekly emails, and the REDCap database online questionnaire. Patients’ experiences with the IT solutions were divided into two sub-areas, adherence-increasing and adherence-decreasing experiences.

When positive experiences with the IT solutions were obtained, the patients gained motivation for adherence to the intervention. Especially when the IT solution was perceived as simple to use, easily accessible and time-saving, the patients were not only persistent in using it but also gained a positive attitude towards the content of the IT solution. Furthermore, it was perceived as an advantage if the informants could choose for themselves when, how and where to access the intervention.

Informant number 9:

So, the functionality of the emails is very, very easy. So it’s just a matter of clicking on the link and reading the email and confirming that you have read it, so it’s completely problem-free. And it also helps making me read them….

The patients said that the visual graphs made it easier for them to grasp the possible benefit from a behaviour change.

Informant number 7:

I’d rather have the graphs than I want a number. Because then you can really see it.

Informant number 6:

“And there she (the health care worker) also showed in connection with the fact that I had stopped smoking, how much it changed on the graph there. So, it was a really positive experience to get through. …Well, it means many years in the end.”

Patients experienced non-functioning IT solutions as a major obstacle. Specific points addressed were problems logging in to the software, problems getting help from IT-service and a simple lack of competences in using the IT solution in the intervention. They were not able to part the negative feelings of the non-functioning IT, from the general perception of participation in the trial. As a result, a non-functional IT solution can remove a patient’s motivation for adherence to the rest of the study, even though the initial problem is solved.

Informant number 2:

“I had a lot of problems with that login and that program in the beginning….

… So, there I quickly lost interest in it because I simply did not get the help, I needed….

… And I also did not answer quite a few of the questionnaires simply because I thought it was sloppy and unprofessional that I could not get help at the beginning, and make use of it, as I understood it should be.”

#### Communication

From the focus area communication, we identified 4 sub-areas: (a) concerns of becoming ill, (b) visual communication, (c) written text and (d) the messenger.

Concerns of becoming ill: Before going into the trial, we set up an aim to explore whether the patients’ life quality would suffer under the intervention, due to feeling sicker from being continuously reminded of their risk of cardiovascular disease. The view from the informants was unanimous – none felt sicker from the intervention.

Informant number 9:

No, it is not something that has stressed me out, you could say, in relation to illness. No, I do not think so.

Visual communication: All patients said that seeing the graphs of their cardiovascular risk profile, gave them a better understanding of their risk and the predictive development hereof than they previously had had.

Written text: It was clear that the level of difficulty in the language used in the written text was of great importance. Patients with shorter educations (elementary school or vocational education) understood the text and did not see it as a barrier for reading the recurrent emails. Patients with longer educations (short or long higher education) acknowledged that the language was fitted to suit all patients and did not perceive it as condescending. The text used in the intervention was easily understood by all, but a minority of patients perceived the emails as without significance because they already knew the content. Even though some patients perceived the content of the emails to be insignificant, they still thought of the emails as a working nudging tool poking to their subconscious.

Informant number 5:

”Well, but I understand that fine. It was written in reasonably normal Danish. And that was actually quite important, I thought.”

Informant number 9:

“Well, it’s probably as I say, that it somehow creeps in a little under the skin of one anyway, even though “I know that”, then it is still “well, I must also remember that, right.”

The messenger: The informants stated that they saw their doctor as a trusted person and as such, they counted on the doctor to call for action if changes regarding their health were needed.

When making their take on the intervention, patients were affected by their perception of the messenger’s attitude towards the intervention. Patients reflected a lot of the doctor’s attitude towards a problem onto their attitude towards that same problem. As such, patients who experienced a doctor who was committed to the trial and found it important, also gained a positive first impression. Patients who experienced a doctor who was more reluctant towards the intervention, gained an attitude towards the intervention as it being less important. It was clear, that the change in insight from using the visual communication tool, was only possible because a health professional had explained the profile and the graphs to the patients, the first time they saw it.

Informant number 9:

“I mean a doctor is - it’s a trusted person (…) so his attitude means something!”

Informant number 1:

“Yes, I think it has, it matters a lot how you get it presented.”

**Table 3:** Summary of results

Focus areas	Sub-areas	Resulting messages
Change of health behaviour	Motivating experiences	Autonomy to choose when to use intervention
		Feeling of an increase in insight into CVD risk
		Personal health gain from intervention
		Relatedness
		Reminders, continuously good advice, or reassurance of knowledge
	Demotivating experiences	Competing illness needing priority
		Non-committed health care personnel
		Non-functioning IT
Information Technology (IT)	Adherence-increasing experiences	Autonomy in when to use the intervention
		Easy access
		Simple to use
	Adherence-decreasing experiences	Lack of competences in using the IT solution
		Problems getting help from IT service
		Problems logging in to the software
Communication	Concerns of becoming ill	Continuously reminders do not make patients feel sicker, and in most cases, it motivates them to adhere to treatment.
	Visual communication	Easier to see benefits from behavioural change
		Easier to understand risk information when presented visually
	Communication in written text	Didn’t find the content interesting because they knew it already
		Important to use easily understandable language
		Text in the intervention has been easily understandable
		Worked as nudging even though informants knew the content already
	Messenger dependant communication	Feelings and commitment are reflected by patients
		Doctors need to help patients understand risk information and educate them
		Patients trust their doctor to know what is best for them

## Discussion

### Main findings

Our findings indicate that the change to a visual risk communication tool could increase the patients’ perceived insight into their CVD risk. However, determining the most crucial factors influencing adherence remains unclear. While easy accessibility and well-functioning IT solutions are essential, the perception of the intervention’s importance is also significant. This perception of the intervention was highly influenced by the doctor, who was reported as a trusted person in whom the patients placed a lot of confidence to take good care of their health. This became even more important, as patients reported severe comorbidities to be a demotivating factor for adhering to CVD risk lowering interventions. These findings agree with former findings by Bonner et al. [21].

The recurrent reminders helped keep the patients motivated and did not make them feel more concerned about becoming ill. This agreed with previous findings, which showed that reminders increased adherence to medical treatment [22, 23].

### Interpretation

To better understand our findings, we contextualise them within behavioural change theories.

Our results indicate that patients benefit from the intervention at start-up, gaining insight into their CVD risk and establishing an alliance with the health care personnel. Throughout the trial patients benefit from continuous maintenance of relatedness and competences. By providing continued support through recurrent push emails the participants were supported during the maintenance stage of the TTM and had the relatedness used in the SDT strengthened [11, 14]. Importantly, patients retained autonomy as the intervention offered advice rather than directives.

The shift to visual communication, increased the patients’ self-estimated understanding of risk, which aligns with increasing their competences towards managing their risk in the SDT but also aligns with the TPB by enhancing understanding control of risk change and the optimal reachable normal state.

We found that GPs’ commitment was important for patients’ perception of the intervention presented to them which fits former study results [21]. Patients’ attitudes towards the intervention were changed in a negative direction when they experienced their doctor as less committed. This correlates with both SDT and TPB since GPs facilitate all three legs of the SDT through shared decision making as well as ensure the patient’s understanding of chances to adhere to treatment and optimal normal state (from TPB [12]).

Patients trusted the doctors’ competences, which strengthened the patients’ relatedness to the intervention, emphasizing the importance of using the communication tool. However, the patients were demotivated from adhering to CVD risk lowering interventions by competing illnesses and it was therefore of great importance that the GP helped the patients prioritise their efforts. By prioritising patients’ resources in shared decision making, patients’ relatedness is strengthened.

When patients experienced IT malfunctions, they had a serious setback in motivation. This can be explained by the SDT, as the patients had no chance of fixing the IT problems, they were completely stripped of competences to adhere to the intervention.

None of the informants felt more concerned about their own health due to the intervention. This is in accordance with the theories SDT and TTM because the intervention helps patients stay in the maintenance stage of the TTM as well as strengthen all legs of the SDT and stimulate behavioural change following the TPB. In no way does the intervention push the patients towards failure.

### Strengths and limitations

Since this was the first research done in Denmark with this communication tool, interviews were chosen instead of questionnaires. Questionnaires was thought to limit informants’ answers and therefore interviews were chosen to bring forth a better description of the patients’ thoughts and experiences related to the trial and the intervention.

One researcher conducted all interviews and transcriptions continuously, ensuring the optimal transfer of experienced insights.

Regarding the limitations of the study, the interviews were done by phone instead of face to face which eliminated the possibility of reading body language. We assessed that the needed information for this kind of interview was obtainable through the phone and did not need face-to-face interaction. The added information from a face-to-face encounter is highly valued in the doctor/patient setting but was evaluated as expendable for this study.

Introducing the interviewer as a doctor might have influenced informants to appear more adherent, but assurances were given that participation would not impact their normal treatment. The interviewer was not the GP of any of the informants.

The interviews were done when the informants were 6–12 months into the trial. This allowed all of them to have had a substantial experience with the trial’s questionnaires and recurrent emails, but it also meant that it was a while since they experienced the online communication tool together with the health professional. This timespan could have affected the informants’ memory of the online communication tool. Two informants expressed fear of lack of memory of the online communication tool due to the timespan since they were introduced to it, and they had not revisited it at home since.

Due to the strategic selection of informants, both educational levels, geographical areas, ages, and employment status were evenly covered which strengthens the belief in the completeness of our data.

Some of the GPs demonstrated (according to the informants) low commitment, which affected the patients’ perception of and adherence to the trial. This can be seen as reflecting the true width of General Practitioners and was therefore not seen as a limitation by the authors.

### Implications

The first lesson learned was the need to focus on the IT solutions being as simple and accessible as possible. It will probably be beneficial if the IT solution is fully compatible with smartphones as this will most likely strengthen patients’ adherence. Further, efforts should be directed towards ensuring good maintenance of the intervention, since downtime greatly decreases patients’ motivation.

When creating interventions containing written text, using direct and easily understandable language is vital for diverse educational levels.

We should strive to communicate risk visually with graphs reaching years ahead. This will give patients an increased feeling of understanding their own risk and a possibility to discuss prognostic aspects in the time to come. These findings fit previous findings where patients’ motivations and perceptions of risk and interventions are important for adherence [8, 21].

The involvement and commitment of healthcare workers, particularly GPs, are crucial for intervention success. This corresponds with findings by Polinski et al. [24] who found that a pre-established trust between patient and provider increases adherence. The collaboration with GPs should be prioritised in future research and implementation projects.

None of the patients in the study felt more concerned of falling ill after increasing their awareness of their CVD risk. Offering more and more individual risk information should therefore be seen as an advantage rather than negatively affecting patients [8].

## Conclusion

This study highlighted patients’ overall positive attitude toward the intervention while identifying challenges and barriers influencing adherence.

The visual and dynamic communication tool increased motivation by heightening perceived insight into CVD risk without worsening patients’ concerns of illness.

Continuous advice through e-mails was never perceived as demotivating.

When the IT solution worked and was simple and accessible, it increased study participants’ adherence to the intervention, but the opposite was also the case. The success of IT-dependent interventions therefore relies on well-maintained, smoothly functioning IT solutions.

GPs play a pivotal role in shaping patients’ perceptions of interventions, particularly in prioritizing resources amid competing illnesses. Overall, the study emphasizes the importance of a patient-centric approach in implementing interventions for cardiovascular health.

## Data Availability

Data from the interviews (audio recordings and transcriptions) are saved on a secure server at the University of Southern Denmark and are only available to the research team. Data can be made available on reasonable request by contacting the corresponding author.

## Abbreviations

CVD: Cardio-vascular disease
GP: General Practitioner
IT: Information Technology
SDT: Self Determination Theory
TPB: Theory of Planned Behaviour
TTM: Transtheoretical Model Health Behaviour of Change
